# Confocal fluorescence assessment of bioenergy/redox status of dromedary camel (*Camelus dromedarius*) oocytes before and after *in vitro* maturation

**DOI:** 10.1186/1477-7827-12-16

**Published:** 2014-02-18

**Authors:** Roberto Russo, Davide Monaco, Marcello Rubessa, Khalid A El-Bahrawy, Ashraf El-Sayed, Nicola A Martino, Benedicte Beneult, Francesca Ciannarella, Maria E Dell’Aquila, Giovanni M Lacalandra, Manuel Filioli Uranio

**Affiliations:** 1 Section of Clinics and Animal Productions, Department of Emergency and Organ Transplantation (DETO), University of Bari Aldo Moro, Str Prov Casamassima, km 3, Bari Valenzano 70010, Italy; 2 National Research Council (CNR), ISPAAM, c/o Istituto tecnico agrario statale “E. De Cellis”, Via Argine 1085, Naples Ponticelli80147, Italy; 3 A.I. Lab., Maryout Research Station (MRS), Desert Research Center (DRC), Al-Nasryia St., Al Amryia, Alexandria, Egypt; 4 Cairo University Research Park (CURP), Faculty of Agriculture, Cairo University, Giza 12613, Egypt; 5 SupAgro, Systèmes d’Elevage Méditerranéens et Tropicaux, UMR_SELMET, Montpellier 34598, France; 6 Department of Animal Production, Faculty of Agriculture, Cairo University, Giza, Egypt

**Keywords:** Dromedary camel oocyte, *in vitro* maturation (IVM), Mitochondrial distribution pattern, Mitochondrial activity, Intracellular reactive oxygen species (ROS) levels, Mitochondria/ROS colocalization

## Abstract

**Background:**

Reproductive biotechnologies in dromedary camel (*Camelus dromedarius*) are less developed than in other livestock species. The *in vitro* maturation (IVM) technology is a fundamental step for *in vitro* embryo production (IVP), and its optimization could represent a way to increase the success rate of IVP. The aim of the present study was to investigate the bioenergy/oxidative status of dromedary camel oocytes before and after IVM by confocal microscopy 3D imaging.

**Methods:**

Oocytes were retrieved by slicing ovaries collected at local slaughterhouses. Recovered oocytes were examined before and after IVM culture for nuclear chromatin configuration and bioenergy/oxidative status, expressed as mitochondria (mt) distribution and activity, intracellular Reactive Oxygen Species (ROS) levels and distribution and mt/ROS colocalization.

**Results:**

The mean recovery rate was 6 oocytes/ovary. After IVM, 61% of oocytes resumed meiosis and 36% reached the Metaphase II stage (MII). Oocyte bioenergy/redox confocal characterization revealed changes upon meiosis progression. Immature oocytes at the germinal vesicle (GV) stage were characterised by prevailing homogeneous mt distribution in small aggregates while MI and MII oocytes showed significantly higher rates of pericortical mt distribution organized in tubular networks (P < 0.05). Increased mt activity in MI (P < 0.001) and MII (P < 0.01) oocytes compared to GV stage oocytes was also observed. At any meiotic stage, homogeneous distribution of intracellular ROS was observed. Intracellular ROS levels also increased in MI (P < 0.01) and MII (P < 0.05) oocytes compared to GV stage oocytes. The mt/ROS colocalization signal increased in MI oocytes (P < 0.05).

**Conclusions:**

This study provides indications that qualitative and quantitative indicators of bioenergy and oxidative status in dromedary camel oocytes are modified in relation with oocyte meiotic stage. These data may increase the knowledge of camel oocyte physiology, in order to enhance the efficiency of IVP procedures.

## Background

The dromedary camel (*Camelus dromedarius*) is a seasonal polyestrous breeder and reproductive activity is limited to the coolest month of the year [1]. Local environmental factors such as rainfall, external temperature, and nutrition, rather than photoperiod, are supposed to influence the moment of initiation of sexual activity and length of breeding season [2]. Like other camelids, dromedary camels are induced ovulators [3] but spontaneous ovulation sometimes occurs [4]. The breeding season occurs during winter (November/December to March/April), though there are conflicting reports about its beginning and length [5]. Outside the breeding season, mating activity ceases and the ovaries are inactive or have a few small follicles [5].

The interest in dromedary camel breeding is due to its role as a food resource in semi-arid areas and also to the commercial interest in the racing sector. The total number of camels in the world is reckoned to be around 25 million heads; though this number is probably underestimated, a steady increase has been recorded in the last 20 years [6]. In order to optimize breeding programs in this species, monitoring and increasing efficiency plans of the reproductive activity are desirable. To this end, application of assisted reproductive technologies (ARTs), such as artificial insemination, oocyte *in vitro* maturation (IVM) and fertilization, oocyte and embryo cryopreservation, embryo transfer and cloning could provide a substantial opportunity to improve reproductive efficiency and genetic performance and to better understand factors regulating reproductive activity in this species.

The successful development of ARTs relies on obtaining functionally competent oocytes which are a mandatory requisite for successful embryo production.

IVM is a fundamental step in *in vitro* embryo production (IVP) and, in any species, its optimization could represent a way to increase the success rate of IVP. The regulation of oocyte maturation not only affects the proportion of oocytes capable of undergoing maturation, but also their subsequent fertilization and embryo development [7]. There is very little information on oocyte physiology in camelids, although the kinetics of oocyte nuclear maturation has been studied [8,9]. The complex process of cytoplasmic maturation has not been widely investigated in this species. To date, only one study reported offspring in camelids obtained by transfer of embryos produced by IVP [10]. These authors recovered oocytes from abattoir-derived ovaries, cultured them *in vitro* for 30 h in TCM-199 medium supplemented with fetal calf serum (FCS), epidermal growth factor (EGF), follicle stimulating hormone (FSH), estradiol and cysteamine. After maturation, oocytes underwent *in vitro* fertilization with motile spermatozoa obtained by centrifugation of fresh semen on a Percoll discontinuous gradient and putative embryos were cultured in a semi-defined medium (mKSOMaa supplemented after 48 h with 10% FCS). The same authors reported 23% blastocyst formation rate and 13 normal dromedary camel offspring produced by IVP [10]. Other studies have been reported on IVM technology in dromedary camel oocytes [5,9,11-13]. These studies analysed the role of reproductive season and the effects of different culture media on *in vitro* oocyte meiotic development.

Increasing evidence shows the role of mitochondria (mt) as determinant for developmental competence of human and mammalian oocytes [14-16]. Regular mitochondrial bioenergetic activities within the oocyte include mt adenosine triphosphate (ATP) generation, roles in Ca^2+^ homeostasis, regulation of cytoplasmic redox state, and signal transduction [17,18]. It has been reported that oocyte competence is directly related to cytoplasmic bioenergetic capacity, and that ATP supply/demand imbalances, associated with suboptimal levels of mitochondrial ATP generation, may be a common cause of several developmental defects [15]. Mitochondrial dysfunctions or abnormalities may compromise developmental processes by inducing chromosomal segregation disorders, maturation and fertilization failures, or oocyte/embryo fragmentation resulting in mt-driven apoptosis [15,17]. Mitochondria are the major source of reactive oxygen species (ROS), which are produced during oxidative phosphorylation [19]. Under physiologic conditions, ROS are neutralized by an elaborate defence system [20]. If disequilibrium between ROS production and antioxidative capacity of the cell takes place, oxidative stress occurs [21]. Although the impact of oxidative stress on oocyte and reproductive function remains unclear, it has been shown that oxidative stress affects oocyte Ca^2+^ homeostasis, and thus also oocyte maturation and oocyte-sperm interaction [22].

To our knowledge, only one study has been reported to date on mt complement distribution and activity in dromedary camel oocytes as assessed by confocal microscopy [12]. These authors investigated mt distribution and activity, ATP, glutathione (GSH) content and calcium oscillation during IVM. They observed a highly polarized mt distribution pattern in MII oocytes compared to immature ones. In the same study, higher ATP content was observed in MII oocytes than in immature ones.

The aim of the present study was to investigate the bioenergy/oxidative status of dromedary camel oocytes in relation to their meiotic stage by simultaneous analysis of oocyte nuclear chromatin configuration and qualitative and quantitative aspects of bioenergy/oxidative status. A confocal laser scanning microscopy (CLSM)-based multiparametric assessment of oocyte bioenergy/redox status, expressed as mitochondrial distribution pattern, mitochondrial activity, intracellular ROS levels and localization and mitochondria/ROS colocalization, was performed. Increasing the knowledge of camel oocyte physiology could be useful to enhance the efficiency of IVM procedures, for a species in which reproductive biotechnologies would have wide application.

## Methods

The study was performed in accordance with the ethical standards laid down in the 1964 Declaration of Helsinki and its subsequent amendments. All the procedures with animals were performed following good veterinary practice for animal welfare according to the Italian law (D.Lgs 116/92).

### Chemicals and drugs

All chemicals for *in vitro* cultures and analyses were purchased from Sigma-Aldrich (Milan, Italy) unless otherwise indicated.

### Oocyte collection and maturation *in vitro*

Oocyte collection and *in vitro* culture were performed in Egypt. Five replicates were performed during the early non breeding season (May-June 2012 and 2013). Ovaries were collected at a local abattoir, transported to the laboratory in physiological saline solution at 30–35°C and washed once in 70% ethanol and twice with warm (37°C) phosphate buffered saline (PBS) containing 100 IU/mL penicillin G sodium and 100 μg/mL streptomycin. Cumulus-oocyte complexes (COCs) were recovered by slicing ovaries as previously reported [23]. Oocytes with uniform cytoplasm and multilayered cumulus cells were selected, washed twice in HEPES-TCM medium: TCM 199 supplemented with 25 mM HEPES, 2 mM sodium bicarbonate, 2 mM sodium pyruvate, 1 mM L-glutamine and 10% FCS and destined to either immediate evaluation or *in vitro* maturation (IVM) culture. The IVM culture was performed in TCM199 (Gibco 22340) supplemented with 15% FCS, 0.25 mg/mL pyruvic acid, 20 ng/mL EGF, 50 μg/mL gentamicin, 10 μg/mL FSH (Sigma-Aldrich, F2293), 10 μg/mL luteinizing hormone (LH; Sigma-Aldrich, L6420), 1 μg/mL estradiol and 0.3 mM cystine. Groups of 25 COCs were matured in 400 μl of IVM medium, covered with 400 μl of mineral oil, in four well culture plates (Nunc, Roskilde, Denmark). On the basis of previously reported studies, oocytes were cultured for 40–42 h at 38.5°C under 5% CO_2_ in air with 95% relative humidity [12,24]. Oocytes were denuded by gentle pipetting in HEPES-TCM 199 with 80 IU of hyaluronidase and underwent epifluorescence and confocal microscopy evaluation.

### Oocyte mitochondria and ROS staining

To investigate mitochondrial distribution and apparent energy status, oocytes were washed three times in PBS with 3% bovine serum albumin (BSA) and incubated for 30 min in the same medium containing 280 nM MitoTracker Orange CMTM Ros (Molecular Probes M-7510, Oregon, USA) at 38.5°C under 5% CO_2_[25,26]. The cell-permeant probe contains a thiol-reactive chloromethyl moiety. Once the MitoTracker probe accumulates in the mitochondria, it can react with accessible thiol groups on peptides and proteins to form an aldehyde-fixable conjugate. This cell-permeant probe is readily sequestered only by active mitochondria [27,28]. The organelle-specificity of the probe was assessed, as reported by Valentini et al. [29]. Control oocytes were imaged after incubation in MitoTracker Orange and further incubation for 5 min in the presence of 5 μM of the mt membrane potential (Delta Psi)-collapsing uncoupler carbonyl cyanide 3-chloro phenyl hydrazone (CCCP; Molecular Probes; Monza, Italy). This molecule inhibits mt respiratory activity, thus reducing fluorescence intensity. After incubation with mt probe, oocytes were washed three times in PBS with 0.3% BSA and incubated for 15 min in the same media containing 10 μM 2′,7′-dichlorodihydrofluorescein diacetate (H_2_DCF-DA) [30-32] in order to detect and localize intracellular sources of ROS. The principle underlying this procedure may be described briefly as follows: non-ionized H_2_DCF-DA is membrane-permeant and is therefore able to diffuse readily into cells. Once within the cell, the acetate groups are hydrolysed by intracellular esterase activity forming 2′,7′-dichlorodihydrofluorescein (H_2_DCF) which is polar and thus trapped within the cell. H_2_DCF fluoresces when it is oxidized by H_2_O_2_ or lipid peroxides to yield 2′,7′-dichlorofluorescein (DCF). The level of DCF produced within the cells is related linearly to that of peroxides present and thus its fluorescent emission provides a measure of the peroxide levels [30]. After incubation, oocytes were washed three times in pre-warmed PBS without BSA and fixed overnight at 4°C with 2% paraformaldehyde solution in PBS.

### Nuclear chromatin evaluation of oocytes

To evaluate nuclear chromatin, oocytes were stained with 2.5 μg/mL Hoechst 33258 in 3:1 (v/v) glycerol/PBS and mounted on microscope slides covered with cover slips, sealed with nail polish and kept at 4°C in the dark until observation. Oocytes were evaluated in relation to their meiotic stage under an epifluorescence microscope (Nikon Eclipse 600; Nikon Instruments, Amsterdam, The Netherlands; 400x magnification) equipped with B-2 A (346 nm excitation/460 nm emission) filter, as germinal vesicle (GV), metaphase I (MI) and metaphase II (MII) with 1st polar body extruded. Oocytes with multipolar spindle, irregular chromatin distribution or absence of chromatin were classified as degenerated and were excluded from further analysis.

### Assessment of oocyte mitochondrial distribution pattern and intracellular ROS localization

For evaluation of mt distribution pattern, oocytes were selected among those having homogeneous cytoplasmic texture. Oocytes were observed at 600x magnification in oil immersion with Nikon C1/TE2000-U laser scanning confocal microscope (Nikon Instruments, Amsterdam, The Netherlands). A helium/neon laser ray at 543 nm and the G-2 A filter (551 nm exposure and 576 nm emission) were used to point out the MitoTracker Orange CMTM Ros. An argon-ion laser ray at 488 nm and the B-2 A filter (495 nm exposure and 519 nm emission) were used to point out the DCF. For each oocyte, scanning was conducted with 25 optical series from the top to the bottom of the oocyte with a step size of 0.45 μm to allow three-dimensional distribution analysis. General criteria for definition of oocyte mt patterns were adopted on the basis of previous studies in other species [25,26,29,32]. Homogeneous/even distribution of small mt aggregates (SA) throughout the cytoplasm was considered as indication of immature cytoplasmic condition, whereas heterogeneous/uneven distribution of small and/or large aggregates within the cytoplasm indicated a metabolically active ooplasm. In particular, tubular mt networks spread throughout the cytoplasm (diffused tubular network, DTN) or their accumulation in the peripheral cytoplasm (pericortical tubular network, PCTN) or in the periphery and around the nucleus (perinuclear/pericortical tubular network, PN/PCTN) were considered as sequential aspects of the developmental program of cytoplasmic maturation [33]. Oocytes showing irregular distribution of large mt clusters unrelated to the specific cell compartments were classified as abnormal and were excluded from further quantification analysis.

### Quantification of MitoTracker orange CMTM Ros and DCF fluorescence intensity

In each individual oocyte, MitoTracker Orange CMTM Ros and DCF fluorescence intensities were measured at the equatorial plane, as in previous studies in human [34] and animal oocytes [25,26,29,32], with the aid of the EZ-C1 Gold Version 3.70 image analysis software platform for Nikon C1 (Nikon Instruments) confocal microscope. A circle of area = 100 in diameter (arbitrary value) was drawn to measure only the cytoplasmic area (512 by 512 pixels). The fluorescence intensity encountered within the programmed scan area was recorded and plotted against the conventional pixel unit scale (0–255). Fluorescence intensity was expressed as Arbitrary Densitometric Units (ADU). Parameters related to fluorescence intensity were maintained at constant values for all measurements. In detail, images were taken under fixed scanning conditions with respect to laser energy, signal detection (gain) and pinhole size.

### Oocyte mitochondria/ROS colocalization analysis

Colocalization analysis of mitochondria and ROS was performed by using the EZC1 Gold Version 3.70 software. Degree of colocalization was reported as a Pearson’s correlation coefficient quantifying the overlap degree between MitoTracker Orange CMTM Ros and DCF fluorescence signals [26,35].

### Statistics

Nuclear maturation rates and mt distribution patterns were compared by chi-square analysis. For confocal quantification analysis of mt activity and intracellular ROS levels, the least-square means of the dependent variable (MitoTracker Orange CMTM Ros and DCF fluorescence intensity) were calculated in examined samples and the statistical significance of the least-square means between treated and control groups was calculated by one-way ANOVA followed by Multiple Comparison Dunn’s method (SigmaPlot software). For mt/ROS colocalization, mean values of Pearson’s correlation coefficient were compared between treated and control groups by one-way ANOVA followed by Multiple Comparison Dunn’s method (SigmaPlot software). Differences with P < 0.05 were considered as being statistically significant.

## Results

**Figure 1:**
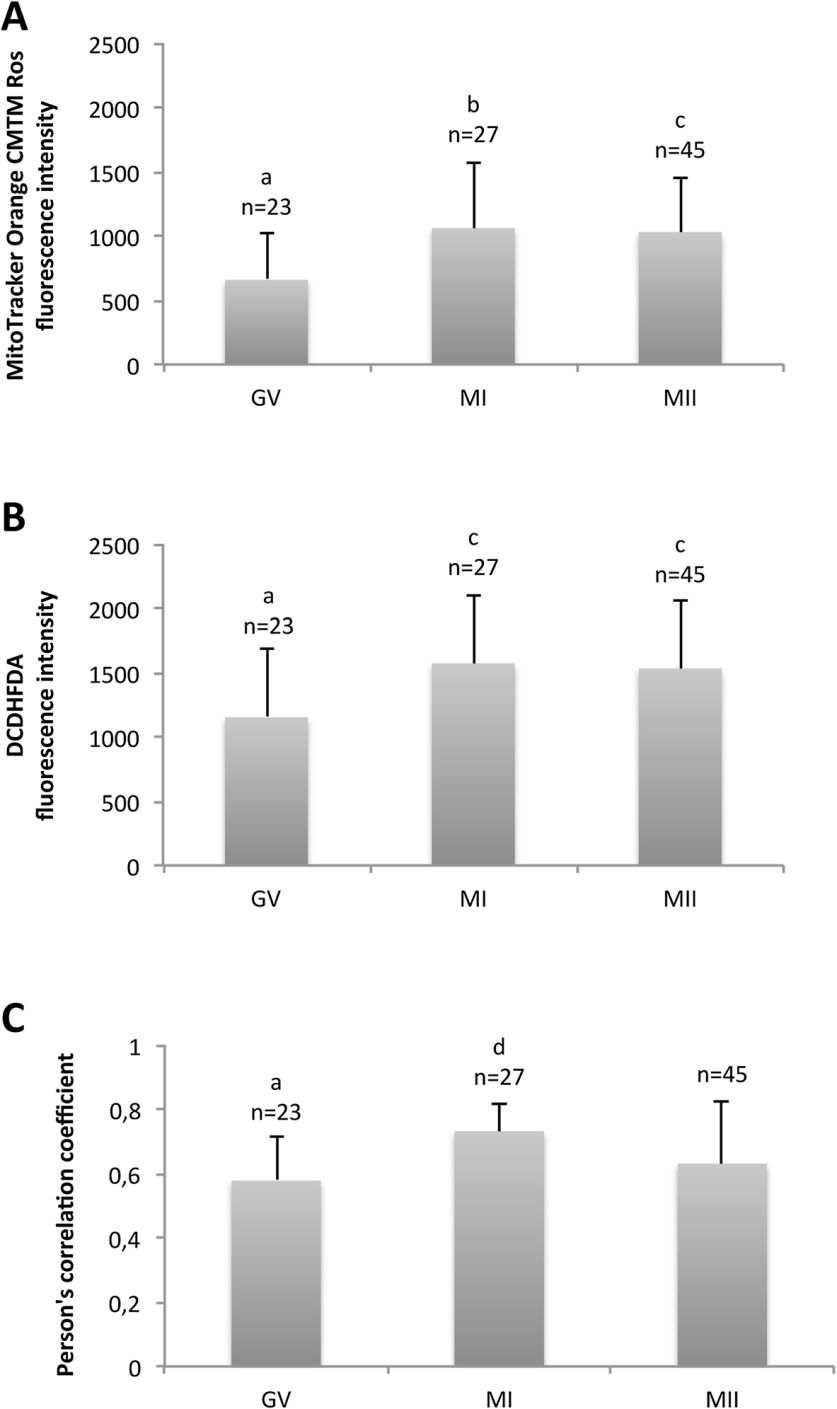
**Quantitative analysis of bioenergy/redox parameters in dromedary camel oocytes before and after *in vitro *maturation.** Mean ± SD of mitochondrial (mt) activity **(Panel A)**, intracellular ROS levels **(Panel B)** and mt-ROS colocalization **(Panel C)** of dromedary camel oocytes, as examined before and after *in vitro* maturation. Numbers of analyzed oocytes per group are indicated above the histograms. Mitochondrial activity and ROS levels are expressed as MitoTracker Orange CMTM Ros fluorescence intensity and 2′,7′-dichlorodihydrofluorescein diacetate (H_2_DCF-DA) fluorescence intensity in Arbitrary Densitometric Units (ADU). Mt/ROS colocalization is expressed as Pearson’s correlation coefficient of MitoTracker Orange CMTM Ros and DCF fluorescent labelling. One-way ANOVA: a,b: P < 0.001; a,c: P < 0.01; a,d: P < 0.05.

After retrieval, 23 oocytes were immediately stained for epifluorescence/confocal analysis. All of them were found at the GV stage and further analysed for bioenergy/oxidative status. One-hundred sixty-five oocytes underwent IVM and after denuding were stained and analyzed. After IVM, 41 oocytes were at the MI stage (25%) and 59 oocytes reached the MII stage (36%). All of them underwent confocal analysis. Data from 23 out of 23 GV stage oocytes, 27 out of 41 MI stage oocytes and 45 out of 59 MII stage oocytes, showing normal ooplasmic size and texture, are reported in Table 1 and Figure 1. Remaining oocytes, which were found at the GV stage (n = 34, 20%) or showing degenerated chromatin configurations (n = 31, 19%) after IVM culture, were excluded from confocal analysis.

**Table 1:** Mitochondrial distribution pattern of dromedary camel oocytes before (*) and after IVM

**Nuclear chromatin configuration**	**N° oocytes**	**SA (%)**	**DTN (%)**	**PCTN (%)**	**PN/PCTN (%)**	**Abnormal (%)**
GV^*^	23	18 (78)a	2 (9)	2 (9)e	1 (4)	0
M I	27	11 (41)b	2 (7)	9 (33)f	5 (19)	0
M II	45	17 (38)c	8 (18)	14 (31)f	4 (9)	2 (4)

Chi-square Test (within columns): a,b: P < 0.02; a,c: P < 0.01; e,f: P < 0.05.

GV: Germinal vesicle; M: Metaphase; SA: Small Aggregates; DTN: Diffused Tubular Network; PCTN: Pericortical Tubular Network; PN/PCTN: Perinuclear/Pericortical Tubular Network.

### Mitochondrial distribution pattern

Results of mt distribution pattern analysis are presented in Table 1. The rate of oocytes showing PCTN mt distribution pattern was significantly higher in oocytes cultured *in vitro* and reaching the MI and MII stages compared with uncultured GV stage oocytes (31% *vs* 9% for MII and GV; 33% *vs* 9% for MI and GV, respectively, P < 0.05). The rate of oocytes showing PN/PCTN also increased with maturation, though not statistically significant. Likewise, the rate of oocytes showing mt distribution in SA was significantly reduced both in MI and MII oocytes compared to GV oocytes (41% *versus* 78% for MI and GV, respectively, P < 0.02; 38% *vs* 78% for MII and GV, respectively, P < 0.01).

### Bioenergy/redox status analysis

**Figure 2:**
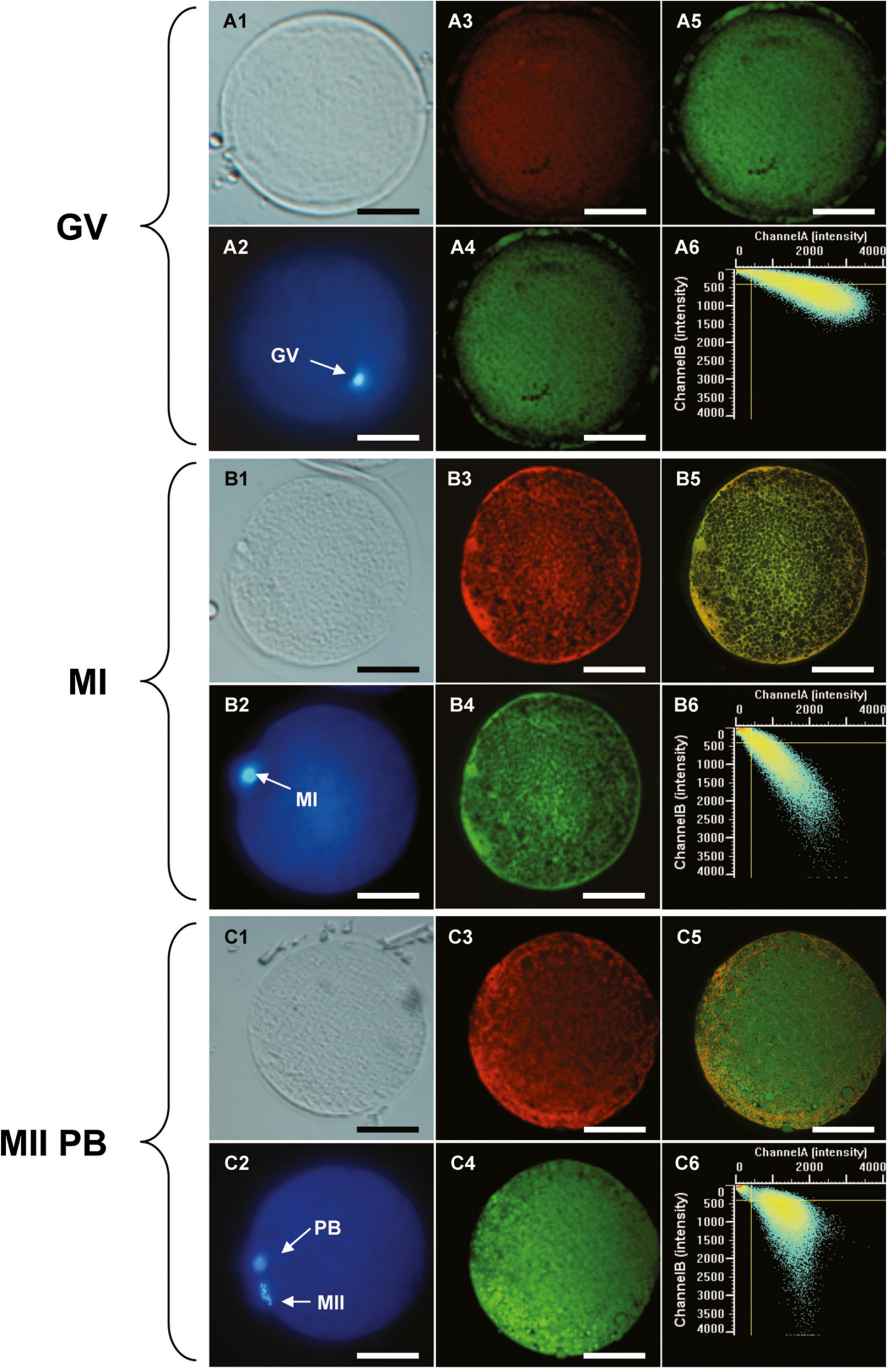
**Confocal images of mt distribution, ROS levels and mt/ROS colocalization of dromedary camel oocytes.** Mitochondrial distribution patterns and intracellular reactive oxygen species (ROS) localization in dromedary camel oocytes at GV **(Panel A)**, MI **(Panel B)**, and MII **(Panel C)** stage observed after staining with MitoTracker Orange CMTM Ros, H_2_DCF-DA and Hoechst 33258. An immature GV stage oocyte showing homogeneous mt distribution pattern of small mitochondrial aggregates **(Panel A)**, a MI oocyte showing heterogeneous distribution of mitochondria (pericortical tubular network, PCTN; **Panel B**), and a MII oocyte showing heterogeneous distribution of mitochondria (perinuclear/pericortical tubular network, PN/PCTN; **Panel C**) are shown. For each sample, corresponding bright field (A1, B1, C1), UV light (A2, B2, C2) and confocal images showing mitochondrial distribution pattern (A3, B3, C3) intracellular ROS localization (A4, B4, C4), mitochondrial/ROS merge (A5, B5, C5) and the mitochondrial/ROS colocalization scatter plot (A6, B6, C6) are shown. The scale bar represents 60 μm.

**Figure 3:**
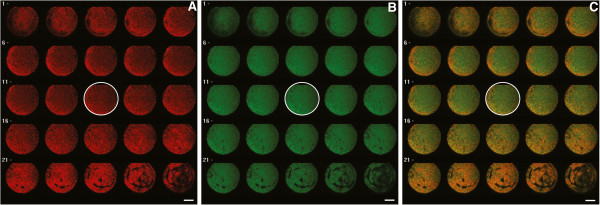
**Twenty-five serial optical sections analysis of bioenergy/redox parameters in a dromedary camel oocyte.** Confocal microscopy twenty-five optical plane analysis of bioenergy/oxidative status in a dromedary camel MII stage oocyte representative of the PN/PCTN mitochondrial pattern. Concerning mt distribution **(A)**, in planes nearer the equatorial position (planes 6–17), the perinuclear/pericortical tubular network (PN/PCTN) pattern can be observed. The section circled in white indicates the equatorial plane which was used for quantification analysis as reported in Figure 1. Values of mitochondria activity **(A)**, intracellular ROS level **(B)** and mt/ROS colocalization **(C)** of the individual oocyte shown in this figure are: 1183 ADU, 1728 ADU and 0.54 Pearson’s coefficient, respectively. Scale bar represents 60 μm.

Significant increases in mt activity in MI and MII oocyte groups compared to GV group (1060.92 ± 478.4 *vs* 659.06 ± 352.5 ADU for MI and GV, respectively; P < 0.001; 1030.01 ± 421.38 *vs* 659.06 ± 352.5 ADU for MII and GV, respectively; P < 0.01) were observed. The same, intracellular ROS levels significantly increased in MI and MII oocyte groups compared to GV group (1571.78 ± 527 *vs* 1152.1 ± 528.96 ADU for MI and GV, respectively; P < 0.01; 1532.9 ± 527.79 *vs* 1152.1 ± 528.96 ADU for MII and GV, respectively; P < 0.05). Mitochondria/ROS colocalization significantly increased in oocytes at the MI stage (0.731 ± 0.088 *vs* 0.578 ± 0.133 for MI and GV, respectively; P < 0.05). In MII oocytes, it tended to be higher although not significantly significant (0.631 ± 0.197 vs 0.578 ± 0.133 for MII and GV, respectively; NS). Figure 1 shows mt activity (Panel A), intracellular ROS levels (Panel B) and mt/ROS colocalization (Panel C) of dromedary camel oocytes as observed before (non-cultured GV stage oocytes) and after IVM (MI and MII stage oocytes). Examples of mt distribution pattern, ROS localization, and mt/ROS co-localization plots of non-cultured GV (Panel A), and in vitro cultured MI (Panel B), and MII (Panel C) stage dromedary camel oocytes are presented in Figure 2. Figure 3 is representative of a 25-optical plane analysis of bioenergy/oxidative status in a MII stage oocyte with PN/PCTN mt pattern.

## Discussion

IVM culture duration could have a significant impact on oocyte metabolic/oxidative status, as expressed by examined confocal microscopy parameters. Wani and Nowshari [24] investigated the optimum time and the kinetics of IVM in the dromedary camel. These authors found that dromedary camel oocytes required 32–44 h of in vitro culture to get an optimum number of oocytes in the MII stage. On this basis, we performed our experiments after 40 h of maturation, which is reported by Wani and Nowshari [24] as the optimum IVM time leading to the highest maturation rate. We analyzed the basal bioenergy/oxidative status of IVM oocytes and how it changes during meiosis progression. Our results on the maturation rate are in line with those reported by other authors [5,12]. Zeidan et al. [5] reported 68.2% maturation rate when using TCM199 in oocytes recovered during the breeding season. In the same study, the maturation rate decreased to 44.4% in oocytes recovered in the non-breeding season. Thus, due to our experimental period (early non-breeding season), our maturation rate (36%) are in line with those reported by these authors for oocytes recovered during the non-breeding season. Other studies reported similar [12] or higher maturation rates (84%, [13] and 81%, [9]) as related to the use of different culture media. Abdoon et al. [12] reported a MII rate of 37.5% by using TCM-199 supplemented with 10% FCS, 10 μg/mL FSH, 10 ng/mL EGF, 10 IU/mL equine chorionic gonadotropin (eCG), and 10 IU/mL human chorionic gonadotropin (hCG). Khatir et al. [13] obtained a maturation rate of 84% by using TCM-199 supplemented with 10% FCS, 10 ng/ml EGF and 250 μM cysteamine. Wani and Wernery [9] reported that 81% of *in vitro* cultured oocytes reached the MII stage by using TCM-199 supplemented with 0.4% bovine serum albumin, 10 μg/mL FSH, 10 μg/mL LH, and 1 μg/mL estradiol.

Regular mt bioenergetic activities within the oocyte play a crucial role as determinants for developmental competence for human and mammalian oocytes [15,16]. During oocyte maturation and fertilization, nuclear changes are coordinated with movements of organelles, in particular mitochondria, and with biochemical changes in the cytoplasm to ensure normal embryo development [36]. Of the numerous cytoplasmic changes occurring during oocyte maturation, the positioning of mitochondria may be involved in concentrating ATP or calcium or in regulating pH in specific regions of the oocyte to support normal developmental processes. A mt distribution pattern polarized in the peri-spindle (perinuclear) and in the pericortical region of the oocyte has been regarded as one aspect of cytoplasmic maturation in different species, as reported in previous studies from our unit in equine, ovine, canine and human oocytes [25,26,29,32,34]. In the present study, qualitative analysis of mt distribution in dromedary camel oocytes, performed before or after *in vitro* maturation, revealed that mt distribution patterns within the oocytes were changed in relation to the meiotic stage. Oocytes at the GV stage showed a predominantly uniform mt distribution, organized in SA. In oocytes at the MI and MII stages, a reduction in oocytes showing SA mt distribution pattern and an increase in oocytes with a heterogeneous PCTN mt distribution pattern were observed. These results are in line with those reported in previous studies. Using confocal analysis, Abdoon et al. [12] reported mt clustering in MI oocytes (referred as matured oocytes without PB in their study). These authors also reported highly polarized mt distribution pattern in the subcortical region of MII oocytes showing the first PB extruded. Using ultrastructural TEM analysis, Kafi et al. [8] reported arrangement of most mitochondria in the oocyte periphery at 24–36 hours IVM culture, which is demonstrated as being the time of MI to MII transition.

To our knowledge, this is the first study jointly evaluating the nuclear chromatin configuration and the bioenergy and redox status of dromedary camel oocytes, with a triple staining procedure and an epifluorescence/3D confocal evaluation of individual oocytes, in relation with meiotic progression. Moreover, the quantification study was performed by objective multiparametric analysis. MitoTracker Orange CMTM Ros fluorescence intensity analysis revealed an increase of mt activity in oocytes at MI and MII stage compared with those at GV stage. This finding indicated that the achievement of nuclear maturation was related to an increase in the number of actively respiring mitochondria within the ooplasm and/or to an increase in the number of active sites per each mitochondrion, as well as to a change in mt distribution. These data are in line with observations by Abdoon et al. [12], concerning increased ATP content in MII compared with GV oocytes. DCF fluorescence intensity also revealed an increase in intracellular ROS levels in oocytes at MI and MII compared to GV stage. This result could be explained in terms of increased oxidative phosphorylation which may be translated into an increase in ROS production.

Mitochondria/ROS colocalization, objectively expressed as Pearson’s correlation coefficient, was higher in oocytes at MI stage compared with GV stage oocytes. It also tended to remain at higher values in MII compared with GV stage oocytes, though not significantly different. Colocalization of ROS and actively respiring mitochondria was reported to be indicative of higher ATP turnover resulting from a more intense mt activity and thus indicative of healthy cell conditions, as demonstrated in hepatocytes [37]. Another study, in mouse oocytes, reported that the regions within the oocyte producing high levels of ROS colocalized with the active mitochondria, as visually assessed by using confocal microscopy, in the majority of *in vivo* MII ovulated oocytes [38]. Moreover, higher levels of mt/ROS colocalization were reported as a reliable marker of *in vivo* matured MII ovine oocytes [26].

## Conclusions

In conclusion, this study provides simultaneous information on bioenergy and redox status of dromedary camel oocytes as related to their meiotic stage before and after *in vitro* maturation and contributes to knowledge of camel oocyte physiology, which may enhance the efficiency of IVM procedures and *in vitro* production of embryos in this species.

## Competing interests

The authors declare that they have no competing interest.

## Authors’ contributions

MED, DM and GML conceived the study. DM, MR, BB and FC performed dromedary camel oocyte collection, *in vitro* maturation and mt/ROS stainings. KAE and AES coordinated ovary recoveries and oocyte collection. RR, BB and FC performed epifluorescence chromatin configuration analysis. RR, MFU and NAM performed confocal analysis and statistical evaluations. MED and GML coordinated confocal analysis and statistical evaluations. MFU, RR and MED wrote the manuscript. All authors read and approved the manuscript.

## References

[B1] Skidmore JA Reproductive physiology in female Old world camelids Anim Reprod Sci 2011 124 148 154 10.1016/j.anireprosci.2010.08.023 20888154

[B2] Fatnassi M Padalino B Monaco D Khorchani T Lacalandra GM Hammadi M Evaluation of sexual behavior of housed male camels (Camelus dromedarius) through female parades: correlation with climatic parameters Trop Anim Health Prod 2014 46 313 321 10.1007/s11250-013-0489-x 24122649

[B3] Marie M Anouassi A Induction of luteal activity and progesterone secretion in the non-pregnant one-humped camel (Camelus dromedarius) J Reprod Fertil 1987 80 183 192 10.1530/jrf.0.0800183 3598953

[B4] Nagy P Juhasz J Wernery U Incidence of spontaneous ovulation and development of the corpus luteum in non-mated dromedary camels (Camelus dromedarius) Theriogenology 2005 64 292 304 10.1016/j.theriogenology.2004.11.020 15955354

[B5] Faye B Bonnet P Johnson EH Camel sciences and economy in the world: current situation and perspectives Proceeding of the 3rd Conference of the international Society of Camelid Research and Development. 29th Jan-1st Feb 2012, Muscat, Sultanate of Oman 2012 2 15

[B6] Zeidan AEB El-Harairy MA Gabr SA Tag El-Dien MA Abd El-Rahman SA Amer AM In vitro maturation of camel oocytes as affected by different media during breeding and Non-breeding seasons J Ame Sci 2011 7 460 472

[B7] Bavister BD Rose-Hellekant TA Pinyopummintr T Development of in vitro matured/in vitro fertilized bovine embryos into morulae and blastocysts in defined culture medium Theriogenology 1992 37 127 146 10.1016/0093-691X(92)90251-L

[B8] Kafi M Mesbah F Nili H Khalili A Chronological and ultrastructural changes in camel (Camelus dromedarius) oocytes during in vitro maturation Theriogenology 2005 63 2458 2470 10.1016/j.theriogenology.2004.09.059 15910926

[B9] Wani NA Wernery U In vitro maturation of dromedary (Camelus dromedarius) oocytes: effect of different protein supplementations and epidermal growth factor Reprod Dom Anim 2010 45 e189 e193 10.1111/j.1439-0531.2008.01198.x 20088850

[B10] Khatir H Anouassi A The first dromedary (Camelus dromedarius) offspring obtained from in vitro matured, in vitro fertilized and in vitro cultured abbatoir-derived oocytes Theriogenology 2006 65 1727 1736 10.1016/j.theriogenology.2005.09.029 16263162

[B11] Nagy P Skidmore JA Juhasz J Use of assisted reproduction for the improvement of milk production in dairy camels (Camelus dromedarius) Anim Reprod Sci 2013 136 205 210 10.1016/j.anireprosci.2012.10.011 23146200

[B12] Abdoon ASS Kandil OM Zeng SM Cui M Mitochondrial distribution, ATP-GSH contents, calcium [Ca2+] oscillation during in vitro maturation of dromedary camel oocytes Theriogenology 2011 76 1207 1214 10.1016/j.theriogenology.2011.05.019 21820723

[B13] Khatir H Anouassi A Tibary A Quality and developmental ability of dromedary (Camelus dromedarius) embryos obtained by IVM/IVF, in vivo matured/IVF or in vivo matured/fertilized oocytes Reprod Dom Anim 2007 42 263 270 10.1111/j.1439-0531.2006.00775.x 17506804

[B14] Eichenlaub-Ritter U Wieczorek M Lüke S Seidel T Age related changes in mitochondrial function and new approaches to study redox regulation in mammalian oocytes in response to age or maturation conditions Mitochondrion 2011 11 783 796 10.1016/j.mito.2010.08.011 20817047

[B15] Van Blerkom J Mitochondrial function in the human oocyte and embryo and their role in developmental competence Mitochondrion 2011 11 797 813 10.1016/j.mito.2010.09.012 20933103

[B16] Wang LY Wang DH Zou XY Xu CMJ Mitochondrial functions on oocytes and preimplantation embryos J Zheijang Univ Sci B 2009 107 483 492 10.1631/jzus.B0820379 PMC2704965 19585665

[B17] Brookes PS Yoon Y Robotham JL Anders MW Sheu SS Calcium, ATP, and ROS: a mitochondrial love-hate triangle Am J Physiol Cell Physiol 2004 287 C817 C833 10.1152/ajpcell.00139.2004 15355853

[B18] Dumollard R Carroll J Duchen MR Campbell K Swann K Mitochondrial function and redox state in mammalian embryos Sem Cell Dev Biol 2009 20 346 353 10.1016/j.semcdb.2008.12.013 19530278

[B19] Cadenas E Davies KJ Mitochondrial free radical generation, oxidative stress, and aging Free Radic Biol Med 2000 29 222 230 10.1016/S0891-5849(00)00317-8 11035250

[B20] Winyard PG Moody CJ Jacob C Oxidative activation of antioxidant defence Trends Biochem Sci 2005 30 453 461 10.1016/j.tibs.2005.06.001 15996871

[B21] Finkel T Holbrook NJ Oxidants, oxidative stress and the biology of ageing Nature 2000 408 239 247 10.1038/35041687 11089981

[B22] Agarwal A Aponte-Mellado A Premkumar BJ Shaman A Gupta S The effects of oxidative stress on female reproduction: a review Reprod Biol Endocrinol 2012 10 49 10.1186/1477-7827-10-49 22748101 PMC3527168

[B23] Hamano S Kuwayama M In vitro fertilization and development of bovine oocytes recovered from the ovaries of individual donors: a comparison between the cutting and aspiration method Theriogenology 1993 39 703 712 10.1016/0093-691X(93)90255-4 16727247

[B24] Wani NA Nowshari MA Kinetics of nuclear maturation and effect of holding ovaries at room temperature on in vitro maturation of camel (Camelus dromedarius) oocytes Theriogenology 2005 64 75 85 10.1016/j.theriogenology.2004.11.009 15935844

[B25] Ambruosi B Lacalandra GM Iorga AI De Santis T Mugnier S Matarrese R Goudet G Dell’Aquila ME Cytoplasmic lipid droplets and mitochondrial distribution in equine oocytes: implications on oocyte maturation, fertilization and developmental competence after ICSI Theriogenology 2009 71 1093 1104 10.1016/j.theriogenology.2008.12.002 19167745

[B26] Martino NA Lacalandra GM Filioli Uranio M Ambruosi B Caira M Silvestre F Pizzi F Desantis S Accogli G Dell’Aquila ME Oocyte mitochondrial bioenergy potential and oxidative stress: within-/between-subject, in vivo versus in vitro maturation, and age-related variations in a sheep model Fertil Steril 2012 97 720 728 10.1016/j.fertnstert.2011.12.014 22260855

[B27] Poot M Zhang YZ Krämer JA Wells KS Jones LJ Hanzel DK Laugade AG Singer VL Haugland RP Analysis of mitochondrial morphology and function with novel fixable fluorescent stains J Histochem Cytochem 1996 44 1363 1372 10.1177/44.12.8985128 8985128

[B28] Torner H Brüssow KP Alm H Ratky J Pöhland R Tuchscherer A Kanitz W Mitochondrial aggregation patterns and activity in porcine oocytes and apoptosis in surrounding cumulus cells depends on the stage of pre-ovulatory maturation Theriogenology 2004 61 1675 1689 10.1016/j.theriogenology.2003.09.013 15019463

[B29] Valentini L Iorga AI De Santis T Ambruosi B Reynaud K Chastant-Maillard S Guaricci AC Caira M Dell’Aquila ME Mitochondrial distribution patterns in canine oocytes as related to the reproductive cycle stage Anim Reprod Sci 2010 117 166 177 10.1016/j.anireprosci.2009.03.008 19372012

[B30] Yang HW Hwang KJ Kwon HC Kim HS Choi K Oh KS Detection of reactive oxygen species (ROS) and apoptosis in human fragmented embryos Hum Reprod 1998 13 998 1002 10.1093/humrep/13.4.998 9619561

[B31] Kuznetsov AV Kehrer I Kozlov AV Haller M Redl H Hermann M Grimm M Troppmair J Mitochondrial ROS production under cellular stress: comparison of different detection methods Anal Bioanal Chem 2011 400 2383 2390 10.1007/s00216-011-4764-2 21336935

[B32] Ambruosi B Filioli Uranio M Sardanelli AM Pocar P Martino NA Paternoster MS Amati F Dell’Aquila ME In vitro acute exposure to DEHP affects oocyte meiotic maturation, energy and oxidative stress parameters in a large animal model Plos One 2011 6 e27452 10.1371/journal.pone.0027452 22076161 PMC3208636

[B33] Torner H Alm H Kanitz W Goellnitz K Becker F Poehland R Bruessow KP Tuchscherer A Effect of initial cumulus morphology on meiotic dynamic and status of mitochondria in horse oocytes during IVM Reprod Domest Anim 2007 42 176 183 10.1111/j.1439-0531.2006.00749.x 17348975

[B34] Dell’Aquila ME Ambruosi B De Santis T Cho YS Mitochondrial distribution and activity in human mature oocytes: GnRH agonist vs antagonist for pituitary downregulation Fertil Steril 2009 91 249 255 10.1016/j.fertnstert.2007.10.042 18367183

[B35] Zinchuk V Grossenbacher-Zinchuk O Recent advances in quantitative colocalization analysis: focus on neuroscience Prog Histochem Cytochem 2009 44 125 172 10.1016/j.proghi.2009.03.001 19822255

[B36] Van Blerkom J Microtubule mediation of cytoplasmic and nuclear maturation during the early stages of resumed meiosis in cultured mouse oocytes PNAS 1991 88 5031 5035 10.1073/pnas.88.11.5031 2052585 PMC51801

[B37] Raval J Lyman S Nitta T Mohuczy D Lemasters JJ Kim JS Behrns KE Basal reactive oxygen species determine the susceptibility to apoptosis in cirrhotic hepatocytes Free Radic Biol Med 2006 41 1645 1654 10.1016/j.freeradbiomed.2006.07.023 17145552 PMC1773006

[B38] Wakefield SL Lane M Schulz SJ Hebart ML Thompson JG Mitchell M Maternal supply of omega-3 polyunsaturated fatty acids alter mechanisms involved in oocyte and early embryo development in the mouse Am J Physiol Endocrinol Metab 2008 2008 294 e425 e434 18073322 10.1152/ajpendo.00409.2007

